# Anti-secretory and anti-proliferative actions of next-generation dual subtype 2 and 5 somatostatin receptor ligands in neuroendocrine tumor models

**DOI:** 10.3389/fonc.2026.1766563

**Published:** 2026-02-27

**Authors:** Francesco Fedeli, Margarita Bistika, Francesco Ascione, Alessandro Marangelo, Fabio L. Guzzi, Jörg Schrader, Alan G. Harris, Natalia S. Pellegata

**Affiliations:** 1 Department of Biology and Biotechnology “L. Spallanzani”, University of Pavia, Pavia, Italy; 2 Institute for Diabetes and Cancer, Helmholtz Munich, Neuherberg, Germany; 3 Joint Heidelberg-Institute for Diabetes and Cancer (IDC) Translational Diabetes Program, Heidelberg University Hospital, Heidelberg, Germany; 4 I. Department of Medicine, University Medical Center Hamburg-Eppendorf, Hamburg, Germany; 5 Holman Division of Diabetes Endocrinology & Metabolism, New York University (NYU) Langone Medical Center, Grossman School of Medicine, New York University, New York, NY, United States

**Keywords:** acromegaly, pancreatic NETs, pituitary adenomas, somatostatin receptor ligands (SRLs), somatostatin receptors (SSTRs)

## Abstract

**Introduction:**

First-generation somatostatin receptor ligands (SRLs) mainly target SSTR2, whereas neuroendocrine tumors (NETs) often express multiple SSTR subtypes, frequently SSTR5. Dual SSTR2/SSTR5 targeting may enhance anti-hormonal and antiproliferative effects. We evaluated five novel dual SSTR2/SSTR5 agonists (SMTR-001 to SMTR-005) in preclinical NET models to assess their anti-secretory and anti-proliferative effects in representative preclinical NET models.

**Methods:**

The human insulinoma-derived NT-3 cell line and the murine AtT-20 corticotroph cell line, both expressing SSTR2 and SSTR5, were treated with 1–50 nM of the novel SRLs or reference agents (octreotide, pasireotide). Insulin and ACTH secretion were quantified by ELISA and cell viability was measured after 72 h (AtT-20) or 5 days (NT-3). A putative lead compound, SMTR-002, was further tested in 3D spheroid cultures of NT-3 cells. Intracellular cAMP modulation was evaluated after forskolin stimulation in AtT-20 cells.

**Results:**

In NT-3 cells, all dual SRLs inhibited insulin secretion (−65% to −95%), with SMTR-002, SMTR-004, and SMTR-005 showing significantly greater inhibition than octreotide at 10 nM. Each compound also reduced cell proliferation (−30% to −44%). In 3D cultures of NT-3 cells, SMTR-002 reduced insulin secretion to a degree comparable to octreotide but, unlike octreotide, significantly decreased cell proliferation. In AtT-20 cells, four novel SRLs significantly reduced ACTH secretion (-11% to -69%), with SMTR-001 and SMTR-004 showing efficacy comparable to pasireotide. SMTR-002 and SMTR-003 demonstrated the greatest antiproliferative effects (−53% and −48% at 10 nM). In AtT-20 cells, SMTR-002 also suppressed forskolin-induced cAMP accumulation more strongly than reference SRLs.

**Conclusion:**

Dual SSTR2/SSTR5 agonists exhibit antisecretory and antiproliferative activity in NET models that was similar or even superior to reference SRLs. These findings support their further development as next-generation SRLs for SSTR2/5-expressing tumors.

## Introduction

Neuroendocrine tumors (NETs) are a heterogeneous group of neoplasms arising from neuroendocrine cells, with the gut, pancreas, lung and pituitary gland being prominent sites of origin. The incidence of NETs has risen steadily in recent years, with latest estimates showing a doubling from 4.6 to 8.2/100000 population over the period from 2000-2021 (1). Improved diagnosis, expanded treatment options, and longer survival have contributed to a continued increase in the prevalence of NETs in the United States, with the estimated 20-year limited-duration prevalence reaching approximately 248, 500 cases by 2021 (2). While much of this increase is attributable to higher rates of identification of early lesions with localized disease, NETs remain a challenging group of tumors to treat. Their indolent growth often means that lesions go undetected until they present late as metastatic disease, frequently in the liver. Apart from the clinical impact of metastatic progression, many NETs secrete bioactive hormones, leading to complex clinical syndromes (3). As curative surgical treatment of metastatic disease is generally not feasible, treatment relies on a combination of chemotherapy, targeted molecular therapy, and -increasingly- radioligand therapy (RLT). Among the various molecular targets identified in NETs, somatostatin receptors (SSTRs), particularly subtype 2 (SSTR2), and SSTR5 have emerged as critical players in both the diagnosis and treatment of NETs (4–8).

For more than 30 years, somatostatin receptor ligands (SRLs) have served as the cornerstone of the medical management of gastroenteropancreatic (GEP) NETs and acromegaly (due to GH-secreting pituitary NET) (9). First-generation SRLs such as octreotide and lanreotide are SSTR2-specific at clinically relevant doses, and reduce hormone secretion, slow tumor growth and decrease symptoms in patients with GEP NETs (10, 11). The second-generation SSA pasireotide has high affinity for SSTR5 moderate affinity for SSTR1/SSTR3, while its affinity for SSTR2 is lower than that of octreotide/lanreotide. As SSTR5 is the predominantly expressed receptor in corticotroph tumors where it mediates the inhibition of ACTH secretion, pasireotide is clinically used for the treatment of Cushing’ disease (12–14). Pasireotide is also effective in the management of patients with acromegaly that are not sufficiently controlled with octreotide/lanreotide (15, 16). In the clinical setting, pasireotide is associated with hyperglycemia and diabetes, due in part to more potent inhibition of insulin secretion via SSTR5 as compared to suppression of glucagon/GH secretion via SSTR2 (17, 18). For patients with acromegaly that are unsuited to treatment with pasireotide, the GH receptor antagonist pegvisomant is an option, although its role is limited to hormonal control and does not control pituitary tumor growth (19). Despite promising preliminary studies, pasireotide was not pursued for the treatment of NETs that progressed on first-generation SRLs (20).

Given the limited number of SRLs approved for clinical use, there are several unmet needs in patients with NETs. As NETs usually simultaneously express SSTRs other than SSTR2, additive anti-hormonal and anti-proliferative effects could be achieved by simultaneously high affinity targeting of SSTR2 and other SSTRs (21, 22). This could have the benefit of increasing progression-free survival in patients with metastatic NETs, particularly given that virtually all NETs eventually progress on current SSTR2-targeting SRLs (23). Also, as SSTR2 expression may be heterogeneous or can be downregulated in metastatic NETs, the availability of high-affinity compounds targeting other SSTRs in addition to SSTR2 could overcome limitations of current therapies and expand treatment options for patients with variable SSTR expression (24–26). Moreover, SSTR2 is a major target for the treatment of NETs using RLT to deliver radiolabeled isotopes to SSTR2-expressing cells (27–29). The heterogeneity of SSTR2 expression and the lack of other SSTR-targeting compounds means that somatostatin-based radiodiagnostic and treatment options in patients with low or SSTR2-negative tumors are currently nonexistent (30).

The development of next-generation SRLs with optimized pharmacology, enhanced receptor selectivity, and improved efficacy in both imaging and therapy marks a promising frontier in the personalized management of NETs. We here describe the first *in vitro* characterization of a series of novel halogenated peptide SRLs with high affinity for both SSTR2 and SSTR5 and demonstrate potent antihormonal and antiproliferative effects in pituitary and pancreatic (pan) NET models.

## Materials and methods

### Cell lines and culture conditions

AtT-20 cells, a murine pituitary corticotroph cell line were purchased from ATCC (CCL-89). These cells can secrete ACTH and show high expression of SSTR2 and SSTR5 (31, 32). NT-3 cells, kindly provided by Dr. Jörg Schrader, were derived from a young male patient with stage IV well-differentiated insulinoma and were cultivated on plates coated with collagen type IV from human placenta (SigmaAldrich, #C7521) (33, 34). Histopathology confirmed insulin expression and the Ki-67 was determined to be 15%–20%. NT-3 cells secrete insulin, express SSTR2, SSTR3, SSTR5 and divide every 11 days (33). The use of human tissue for the generation of cell cultures was approved by the local ethical review board and written informed consent from the patients was obtained prior to surgery (Ärztekammer Hamburg, PV 3548). The present study was conducted in accordance with the Declaration of Helsinki.

Cells were never kept in culture for more than a few passages and were routinely checked for the presence of mycoplasma using the N-GARDE Mycoplasma kit (Euroclone, # EMK090020). Cells were counted using the DeNovix CellDrop automatic cell counter (Diatech Labline) and stained with the trypan blue vital dye (Sigma-Aldrich, #93595) to discriminate between live and dead cells.

### Compounds

SMTR-001, SMTR-002, SMTR-003, SMTR-004, and SMTR-005 were designed and synthesized by Grafton Therapeutics SARL (Switzerland). These five compounds are octapeptides with an amino acid backbone lacking in affinity for SSTR1, SSTR3, and SSTR4 which contain novel halogenated amino acids that have low to sub-nanomolar affinities at the human SSTR2 (IC_50_: 0.37-2.6 nM) and SSTR5 (IC_50_: 0.73-3.41 nM) receptors (personal communication, Grafton Therapeutics). Octreotide and pasireotide were provided by D.B.A. Italia (Italy) and Italfarmaco (Italy), respectively. All compounds were received in powder formulation, reconstituted in DMSO and stored at -20 °C until used.

### Measurement of receptor affinity

During peptide design, only structures lacking affinity (>1000 nM IC_50_) for human SSTR1, SSTR3 and SSTR4 were chosen for further development (data from Grafton Therapeutics) (35, 36). Subsequently, the affinities of the novel SRLs at human SSTR2 (NP_001041.1) and SSTR5 (NP_001044.1) were re-assessed in a competitive radioligand binding assay (Euroscreenfast, Gosselies, Belgium). SSTR 2 and SSTR5 were expressed in CHO-K1 cells and competition binding was performed using ^125^I-somatostatin ST14 as the tracer and somatostatin-28 as the reference competitor. The test peptides were studied across a dose range from 0.03to 100 nM. Nonspecific binding was determined by co-incubation with 200-fold excess of cold competitor. The samples were incubated in a final volume of 0.1 ml and then filtered over filter plates. Filters were washed six times with 0.5 ml of ice-cold washing buffer and 50 µl of Microscint 20 (Packard) were added to each well. The plates were incubated 15 min on an orbital shaker and then counted with a TopCount™ for 1 min/well. Receptor affinities for the five test peptides are shown in **Table 1**.

**Table 1:** Affinities (IC_50_) of five novel SRLs at human SSTR2 and SSTR5.

PEPTIDE NAME	hSSTR2 (IC_50_, nM)	hSSTR5 (IC_50_, nM)
SMTR-001	0.37	1.22
SMTR-002	0.74	0.73
SMTR-003	0.48	0.86
SMTR-004	2.6	3.41
SMTR-005	0.54	0.79

### Enzyme-linked immunosorbent assay

The enzyme-linked immunosorbent assay (ELISA) experiments were conducted using the Mouse/Rat ACTH ELISA Kit (Abcam, #ab263880) to detect ACTH in AtT20 cell experiments, and the Human Insulin (INS) ELISA Kit (Elabscience, #E-EL-H2665) to detect insulin secretion in NT-3 cells as reported (34). For AtT-20 cells complete medium was used and for NT-3 cells serum was omitted. Cell culture supernatants were collected and centrifuged at 1, 300 rpm for 5 minutes to remove cellular debris. The supernatants were immediately processed following the manufacturers’ instructions.

### cAMP-Glo assay

AtT-20 cells were seeded in white-walled 96-well plates (Corning Costar, #3610) in the amount of 3500 cells/well. Drugs were administered to the cells at defined final concentrations for 72 hours. Then, samples were induced with 10 µM forskolin (Sigma-Aldrich, #93049) for 3 hours in induction buffer (IB), which had the following composition: serum-free culture medium (DMEM), 500 μM isobutyl-1-methylxanthine (IBMX; Sigma-Aldrich, #I7018), 100 μM 4-(3-butoxy-4-methoxybenzyl) imidazolidone (Ro 20-1724; Sigma-Aldrich, #B8279). The assay was performed by using the cAMP-Glo Assay kit (Promega Corporation, #V1501) following the manufacturer’s instructions.

### Cell proliferation/viability assay

For 2D cultures of AtT20 and NT-3 cell lines, cells were plated in flat-bottom 96-well plates (Biofil, #TCP011096) using 3500 cells/well for AtT-20 cells, and 20000 cells/well for NT-3 cells. These were treated with reference and experimental compounds at defined final concentrations (1, 2.5, 5, 10, and 50 nM). The duration of treatment was 72 hours for AtT-20 cells and 5 days for NT-3 cells. Cell proliferation was assessed using the WST-1 reagent (Roche, #96992) following the manufacturer’s instructions.

For the 3D culture model, NT-3 cells were grown in a 96-well ultra-low attachment (ULA) round black/clear bottom plate (Corning, #4515). We plated 20000 cells/well for NT-3 cells. After spheroid formation (5 days), cells were treated with octreotide, pasireotide or SMTR-002 at defined final concentrations (1, 2.5, 5, 10, and 50 nM) for 5 days for NT-3 cells, as previously reported (34). Cell proliferation of 3D organotypic cultures was assessed with the CellTiter-Glo 3D viability assay following the vendor’s instructions (Promega, #G7570) and luminescence was recorded with a microplate-reading luminometer.

### Protein isolation and western blotting analysis

Total protein lysates were obtained from cell pellets by using RIPA lysis buffer (Sigma-Aldrich, #R0278) supplemented with PhosSTOP phosphatase inhibitor cocktail (Roche, #4906837001) and cOmplete Mini protease inhibitor cocktail (Roche, #4693159001) and incubated on ice for 30 minutes. Protein quantification was conducted with a bicinchoninic acid (BCA) colorimetric assay using the Pierce BCA Protein Assay Kit (Thermo Fisher Scientific, #23225) with bovine serum albumin (Thermo Fisher Scientific, #23209) as standard. Protein lysates were stored at -80 °C until further use.

Fifty µg of total proteins supplemented with 2X Laemmli Sample Buffer (Bio-Rad, #161-0737) in a 1:1 ratio was loaded on mPAGE 4-12% Bis-Tris Precast Gels (Millipore, #MP8W10) and transferred to nitrocellulose membranes (Bio-Rad, #1620115). Membranes were blocked with 5% BSA (Sigma-Aldrich, #A9647) for 1 hour at room temperature and incubated overnight at 4 °C with SSTR2 and SSTR5-specific primary antibodies (7TM Antibodies, #7TM0356N-IC, #7TM0359N-IC), diluted 1:1.000. Membranes were then incubated with HRP-conjugated secondary antibody (Cytiva Amersham, #NA934), diluted 1:3.000, for 1 hour at room temperature. Protein detection was performed using the SuperSignal™ West Pico PLUS enhanced chemiluminescent system (Thermo Fisher Scientific, #34580). To normalize target protein levels, GAPDH expression was assessed using an HRP-conjugated monoclonal antibody (Santa Cruz Biotechnology, #sc-365062; 1:1, 000 in 5% BSA). The membrane was incubated overnight at 4 °C before signal development.

### Quantitative reverse transcription PCR assay

RNA was extracted from cell pellets with the RNeasy Mini Kit (Qiagen, #74104) and quantified using the Nanodrop 2000 spectrophotometer (Thermo Fisher Scientific, #ND-2000). Samples were stored at -80 °C until further use. Retrotranscription was conducted using the high-capacity RNA-to-cDNA Kit (Thermo Fisher Scientific, #4387406). TaqMan qRT-PCR was performed using the QuantStudio 7 Flex (Thermo Fisher Scientific, #4485701) as reported (34). Primers and probes for human SSTR2, SSTR3 and SSTR5 were as previously reported (34); the mouse genes were: SSTR2 (Thermo Fisher Scientific, #4448892, Mm00436685_g1), SSTR3 (Thermo Fisher Scientific, #4331182, Mm00436695_s1), SSTR5 (Thermo Fisher Scientific, #4331182, Mm01307775_s1). Data were normalized to the housekeeping gene beta-2-microglobulin (B2M). Fold changes were calculated using the 2^-^ΔΔCt method after processing raw Ct values in Microsoft Excel.

### Measurements

Absorbance (WST-1 and ACTH and insulin ELISA assays) and luminescence (CellTiter-Glo 3D and cAMP-Glo assays) were measured using a FLUOstar Omega plate reader (BMG Labtech, #KBS-0024-002).

### Statistical analysis

Statistical analyses were conducted using GraphPad Prism software. Differences between experimental groups were assessed using the analysis of variance (ANOVA). Statistical significance was defined as a p value of less than 0.05. Data are expressed as mean ± standard deviation (SD).

## Results

### Effect of novel dual SRLs on hormone secretion and proliferation of pituitary tumor cells

We evaluated the efficacy of five novel dual SRLs (SMTR-001 to SMTR-005) in AtT-20 mouse corticotroph cells. We confirmed Sstr2 and Sstr5 expression (**Supplementary Figure 1**) and ACTH secretion into cell supernatants, which was unaffected by the presence of serum in the culture medium (**Supplementary Figure 2**). We then treated AtT-20 cells with 5, 10 and 50 nM of the test and reference (octreotide and pasireotide) peptides in complete medium and assessed their effect on ACTH secretion 72 hours post-treatment using a specific ELISA assay.

**Figure 1:**
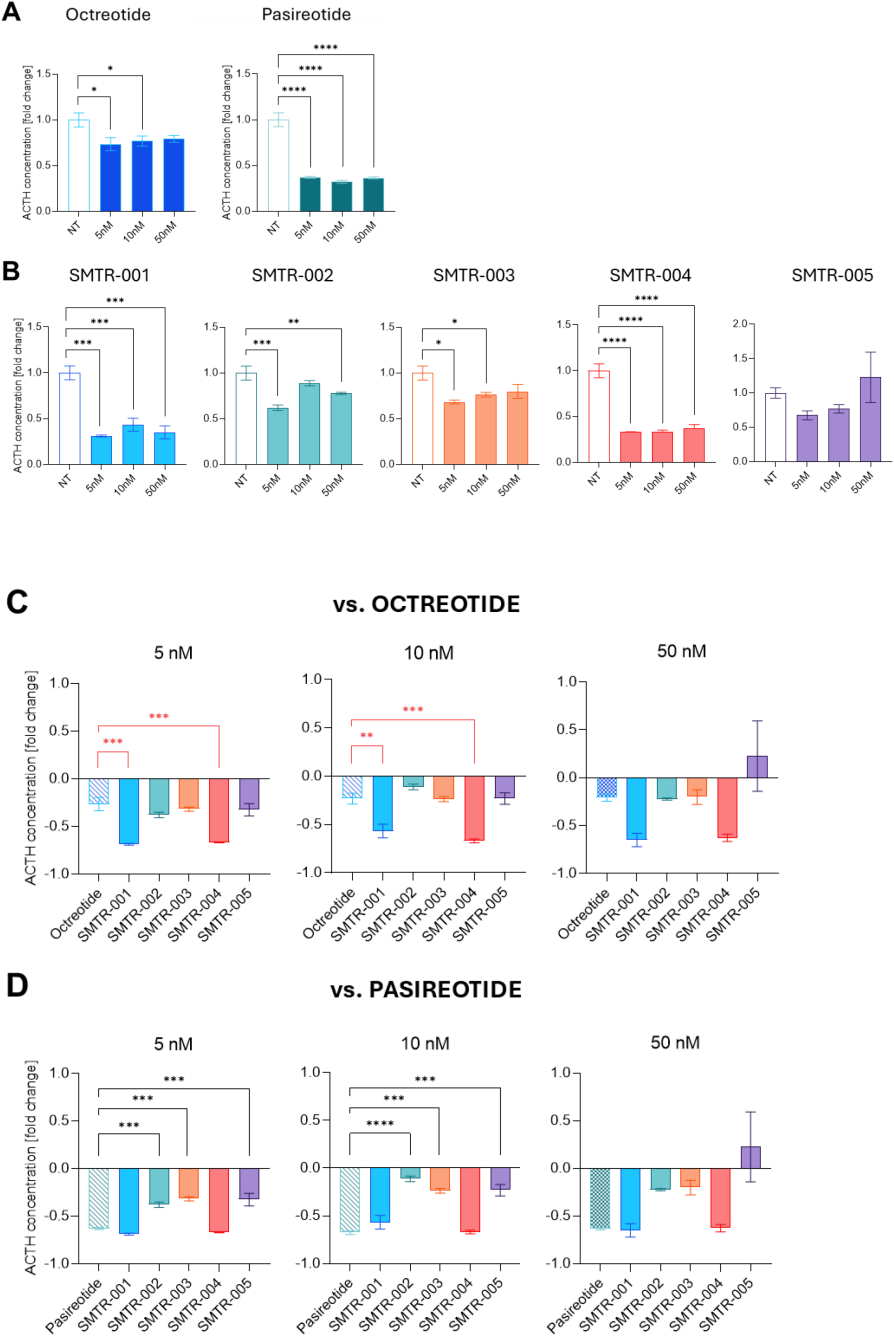
Effect of the test and control SRLs on ACTH secretion in AtT-20 cells. Effect of the test and control SRLs on ACTH secretion in AtT-20 cells. **(A, B)** AtT-20 cells were seeded at a density of 3500 cells/well and then were treated with the indicated concentrations of **(A)** the control SRLs octreotide, pasireotide or **(B)** the test peptides, i.e. 5nM, 10nM, and 50nM. After 72h, supernatants were collected, and ACTH levels were determined by means of the mouse/Rat ACTH ELISA Kit (Abcam). The experiment was repeated 3 times with similar results. Data shown are the average of two technical replicates ± standard deviation (SD). **(C, D)** The decrease in ACTH secretion upon treatment with the test peptides is here compared with that obtained with **(C)** octreotide or **(D)** pasireotide. NT, untreated. In red are the comparisons where the test peptides performed better than the reference drugs, in black are the comparisons where the reference drugs performed better than the test peptides. **(A-D)** Statistical analyses were carried out in GraphPad Prism using one-way ANOVA: *p ≤ 0.05; **p ≤ 0.01; ***p ≤ 0.001; ****p ≤ 0.0001.

Of the five test SRLs, all except SMTR-005, inhibited ACTH secretion at multiple doses, beginning at the lowest concentration tested (5nM) (p ≤ 0.05 to p ≤ 0.0001 *versus* untreated control) (**Figures 1A, B**). Considering the 5nM and 10 nM doses, the inhibitory effects of SMTR-002 (-38% and -11%) and SMTR-003 (-32% and -24%) were equivalent to that of octreotide, while the effects of SMTR-001 (-69% and -57%) and SMTR-004 (-67% at both doses) were similar to those seen with pasireotide (**Figures 1C, D**).

**Figure 2:**
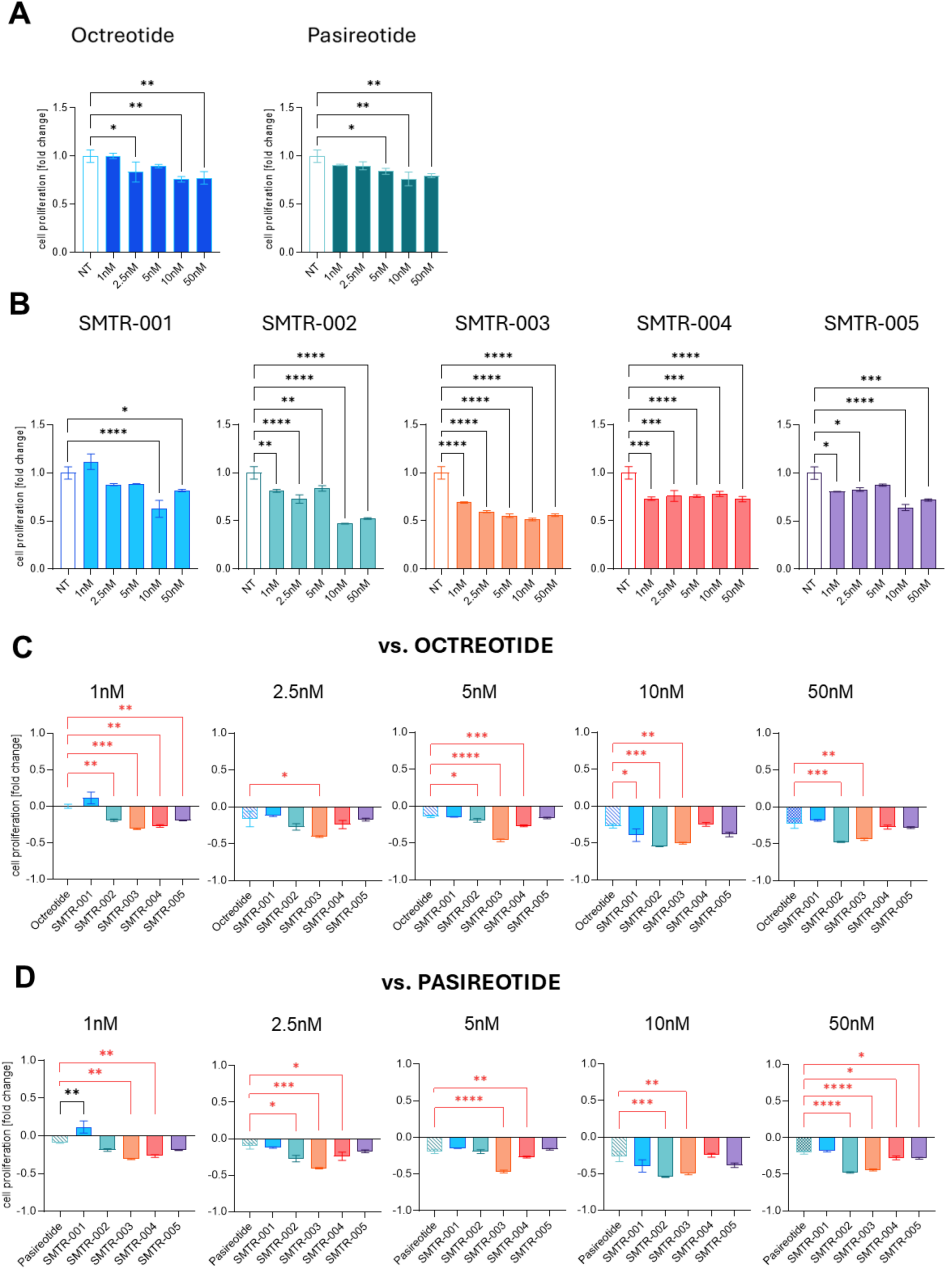
Effect of the test and control SRLs on the proliferation of AtT-20 cells. Effect of the test and control SRLs on the proliferation of AtT-20 cells. **(A, B)** AtT-20 cells were seeded at a density of 3500 cells/well and then were treated with the indicated concentrations (1nM, 2, 5nM, 5nM, 10nM, 50nM) of **(A)** the control SRLs octreotide or pasireotide, or with **(B)** the test peptides, for 72h. Cell viability was then carried out with WST-1 colorimetric assay. The experiment was repeated 3 times with similar results. Data shown are the average of 3 technical replicates ± SD. NT, untreated. **(C, D)** The decrease in cell proliferation upon treatment with the test peptides is here compared with that obtained with **(C)** octreotide or **(D)** pasireotide. NT, untreated. In red are the comparisons where the test peptides performed better than the reference drugs, in black are the comparisons where the reference drugs performed better than the test peptides. **(A-D)** Statistical analyses were carried out in GraphPad Prism using one-way ANOVA: *p ≤ 0.05; **p ≤ 0.01; ***p ≤ 0.001; ****p ≤ 0.0001.

We then evaluated the effects of the test SRLs and reference compounds on AtT-20 cell proliferation after 72 h of incubation. Treatments were applied across a low to mid nanomolar range (1–50 nM) to assess antiproliferative activity (**Figures 2A, B**). Among the test SRLs, SMTR-002 (-53%), SMTR-003 (-48%), SMTR-001 (-37%), and SMTR-005 (-36%) showed clear antiproliferative effects at 10 nM (for all drugs p ≤ 0.0001 *versus* untreated control) (**Figure 2B**). To compare the performance of the test SRLs with the reference drugs at lower doses (1, 5, and 10 nM), their antiproliferative activity was plotted against that of octreotide and pasireotide (**Figures 2C, D**). Across these concentrations, several test SRLs (e.g., SMTR-004) were at least as effective (-25% to -27%) as both reference drugs, and some (e.g., SMTR-002 and SMTR-003) were more effective (-19% to -55% and -31% to -50%, respectively; p ≤ 0.01 to p ≤ 0.001 *versus* untreated control) (**Figures 2C, D**).

**Figure 3:**
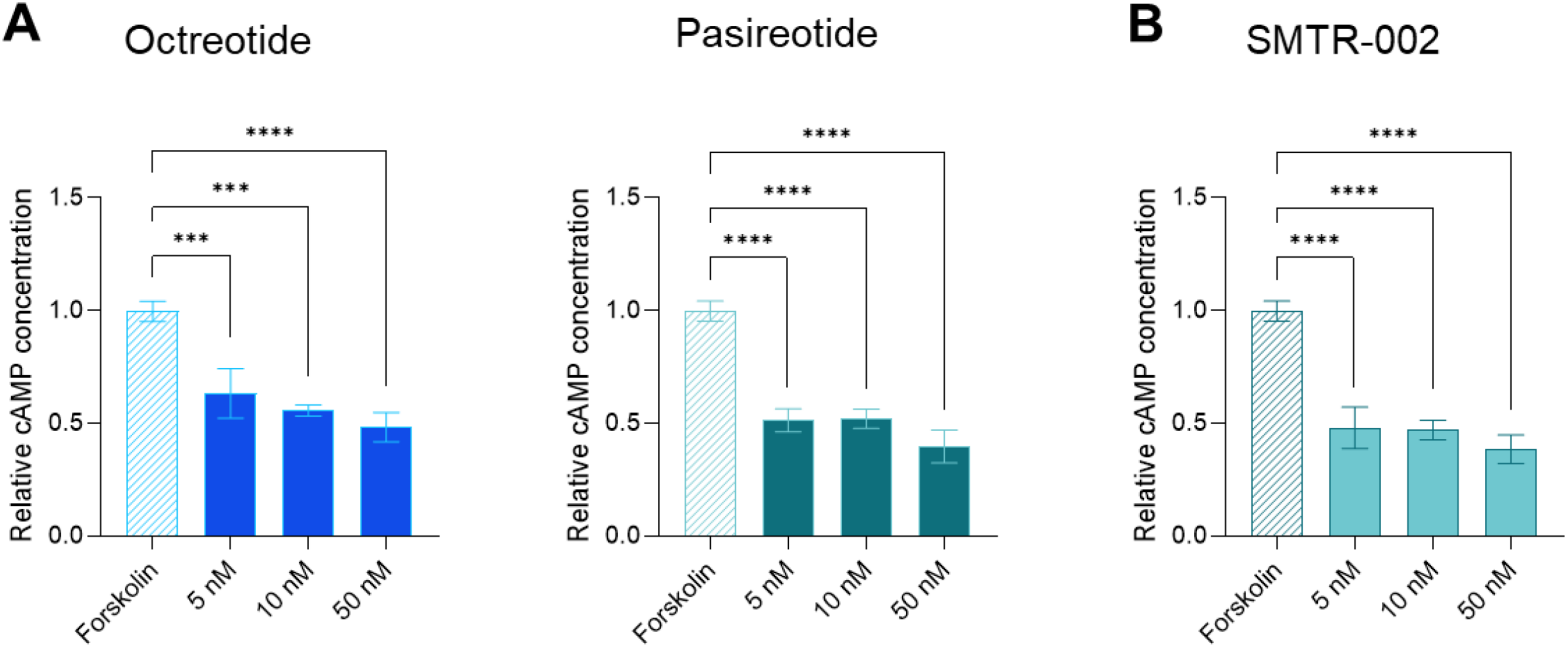
Effect of SMTR-002 and control SRLs on forskolin-induced cAMP production in AtT-20 cells. **(A, B)** AtT-20 cells were seeded at a density of 3500 cells/well and then treated with the indicated concentrations (10nM, 50nM, 100nM) of **(A)** the control SRLs octreotide or pasireotide, or with **(B)** SMTR-002 for 72 hours. Cells were induced with 10 µM forskolin 3 hours prior to performing the assay. The levels of cAMP were then quantified with the cAMP-Glo luminescent assay. The experiment was repeated 3 times with similar results. Data shown are the average of 3 technical replicates ± SD. Data shown are the average of 3 technical replicates ± SD. Statistical analyses were carried out in GraphPad Prism using one-way ANOVA: ***p ≤ 0.001; ****p ≤ 0.0001.

Given that in pituitary cells cAMP is the main functional second messenger that mediates both stimulation and inhibition of hormone secretion, measuring cAMP modulation is directly relevant for assessing SRL effects in pituitary cells (37). It has been shown that exposure of AtT20 cells to octreotide and pasireotide inhibits forskolin-induced cAMP accumulation (37). Thus, we measured forskolin-induced cAMP in AtT-20 cells following a 3-day treatment with the reference drugs (octreotide and pasireotide) and with SMTR-002 for 3 days. SMTR-002 was chosen as a lead peptide in this study because of its equal and subnanomolar affinities at both receptors and for its inhibitory effects on hormonal secretion and proliferation. SMTR-002 was able to reduce intracellular cAMP levels upon forskolin treatment as effectively as pasireotide (-52% to -61%; p ≤ 0.0001 *versus* untreated control at all doses) and already at the 5nM concentration. SMTR-002 inhibited forskolin-induced cAMP levels more strongly than octreotide, especially at the 5nM and 10nM concentrations (p ≤ 0.001 for octreotide and p ≤ 0.0001 for SMTR-002 *versus* untreated control) (**Figures 3A, B**).

### Effect of novel SRLs on insulin secretion and proliferation of human panNET cells

#### 2D culture model

We then evaluated the test SRLs in the NT-3 cell line that is derived from a human panNET (33). NT-3 cells express both SSTR2 and SSTR5 (33, 34; **Supplementary Figure 1**), and retain the ability to secrete insulin (33, 34). As reference agent, we employed octreotide, as it is first line therapy for functioning, well-differentiated panNETs (23).

We first studied the effect of the novel dual SRLs on insulin secretion. Based on the slow proliferation rates of NT-3 cells, we previously determined that a 5-day treatment course was optimal (34). Therefore, we applied this treatment scheme and evaluated the doses of 5, 10 and 50 nM of test and reference SRLs. Insulin levels were measured in the cell supernatant by using a specific ELISA assay.

**Figure 4:**
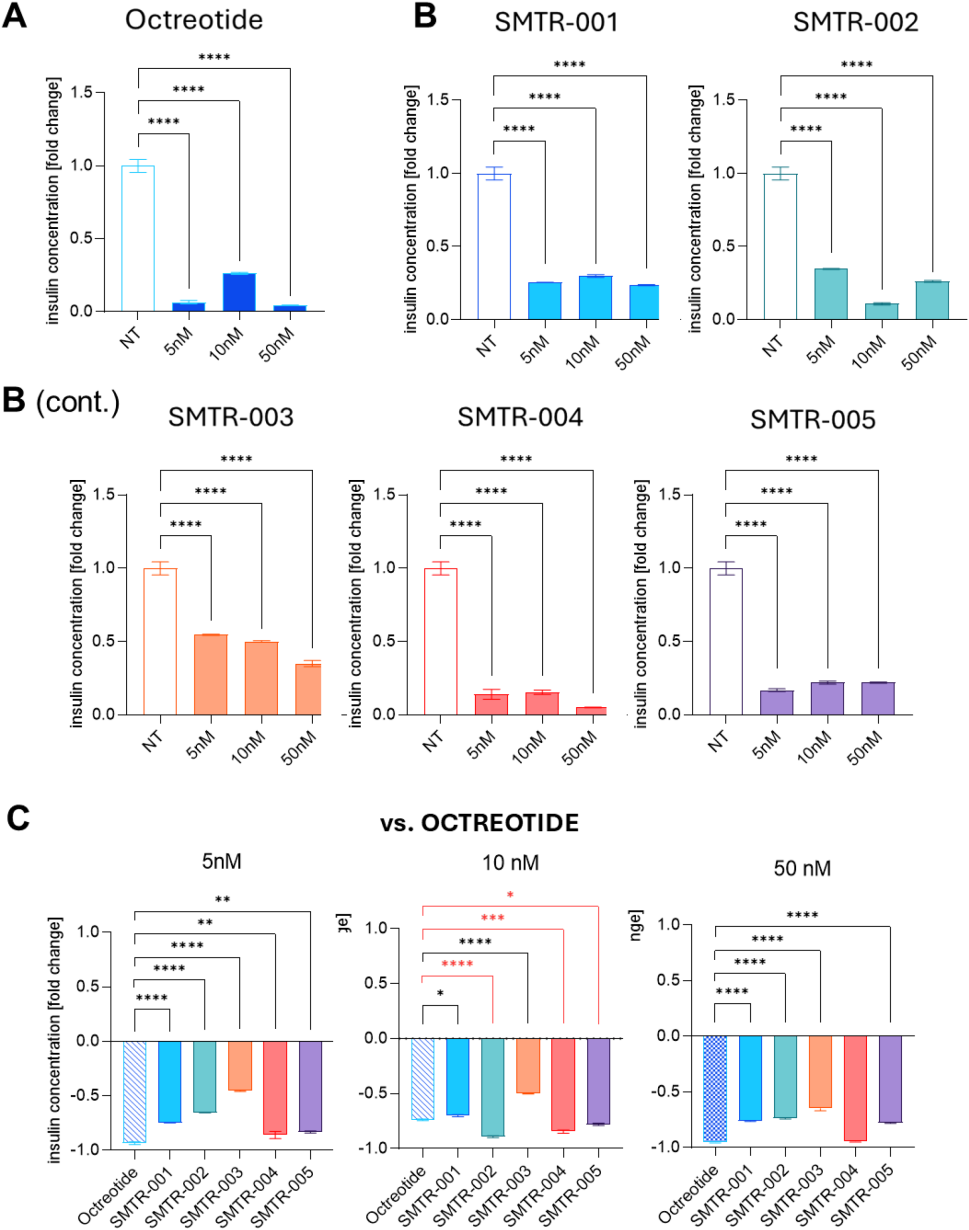
Effect of the test and control SRLs on insulin secretion of NT-3 cells in the 2D culture model. **(A, B)** NT-3 cells were seeded at a density of 20, 000 cells/well and then were treated with the indicated concentrations (i.e. 5nM, 10nM, 50nM) of **(A)** the control SRL octreotide or with **(B)** the test peptides. After 5 days, supernatants were collected, and insulin ELISA was performed using the kit from Elabscience. The experiment was repeated twice with similar results. Data shown are the average of 2 technical replicates ± standard deviation (SD). NT, untreated. **(C)** The decrease in insulin secretion upon treatment with the test peptides is here compared with that obtained with octreotide. In red are the comparisons where the test peptides performed better than the reference drugs, in black are the comparisons where the reference drugs performed better than the test peptides. **(A-C)** Statistical analyses were carried out in GraphPad Prism using one-way ANOVA: *p ≤ 0.05; **p ≤ 0.01; ***p ≤ 0.001; ****p ≤ 0.0001.

When compared with untreated controls, octreotide treatment decreased insulin secretion by -74% to -96% (-45% to -99%; p ≤ 0.001) (**Figure 4A**). All the novel test SRLs significantly inhibited insulin secretion compared with untreated controls (*p* ≤ 0.001). (**Figure 4B**). To better evaluate the effects of the test peptides to reduce insulin secretion *versus* octreotide, we plotted the inhibition of each peptide compared with the reference drug at the lower doses (i.e. 5nM and 10nM). At 10 nM SMTR-002 (*p* ≤ 0.0001 *versus* octreotide) followed by SMTR-004 (*p* ≤ 0.001) and SMTR-005 (*p* ≤ 0.05) were more effective at suppressing insulin secretion than octreotide (**Figure 4C**).

**Figure 5:**
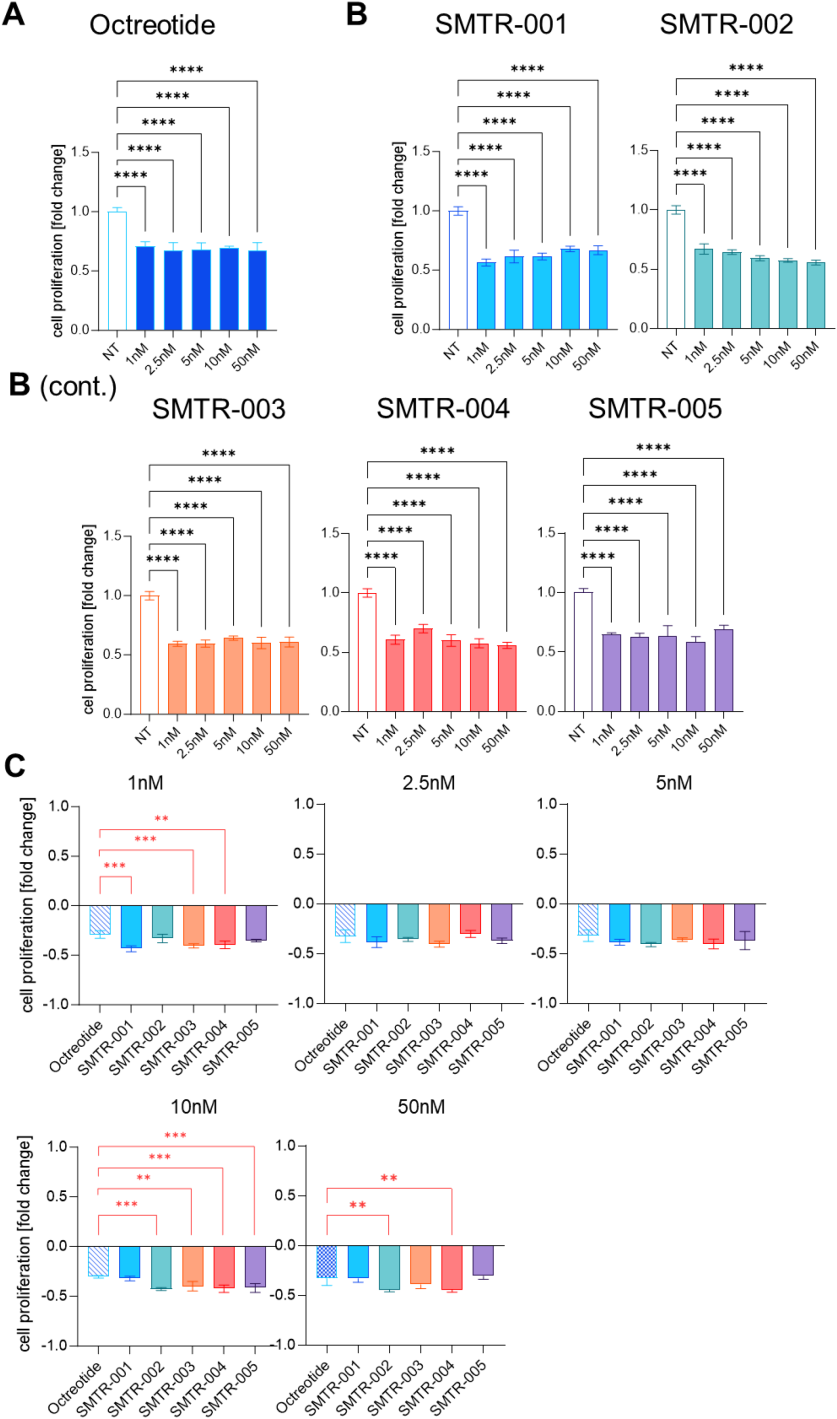
Effect of the test and control SRLs on the proliferation of NT-3 cells in the 2D culture model. **(A, B)** NT-3 cells were seeded at a density of 20, 000 cell/well and then were treated with **(A)** the control SRL octreotide, or with **(B)** the test peptides at the indicated concentrations (1nM, 2, 5nM, 5nM, 10nM, 50nM) for 5 days. Cell viability was then carried out with WST-1 colorimetric assay. The experiment was repeated 3 times with similar results. Data shown are the average of 3 technical replicates ± SD. NT, untreated. **(C)** The decrease in cell proliferation upon treatment with the test peptides is here compared with that obtained with octreotide. In red are the comparisons where the test peptides performed better than the reference drugs, in black are the comparisons where the reference drugs performed better than the test peptides. Statistical analyses were carried out in GraphPad Prism using one-way ANOVA: **p ≤ 0.01; ***p ≤ 0.001; ****p ≤ 0.0001.

We then evaluated the antiproliferative activity of the novel test SRLs *versus* octreotide in NT-3 cells after 5 days of treatment at a dose range of 1nM, 2.5nM, 5nM, 10nM, and 50 nM. All five novel SRLs significantly reduced cell proliferation by 30% to 44% when compared to untreated cells (p ≤ 0.001; **Figures 5A, B**). At the lowest 1nM concentration, the test SRLs SMTR-001 (-43%), SMTR-003 (-40%), SMTR-004 (-39%) inhibited proliferation to a significantly greater extent than octreotide (p ≤ 0.01) (**Figure 5C**).

#### 3D culture model

We then evaluated a lead dual SRL SMTR-002 in 3D organotypic cultures of NT-3 cells, which form viable 3D spheroids in ultra-low attachment plates (Ref. 34; **Supplementary Figure 3A**), whereas AtT-20 cells do not. NT-3 cells in 3D cultures secrete insulin at levels even higher than in 2D cultures (**Supplementary Figure 3B**). This may reflect a more physiological cellular state in the 3D culture model.

**Figure 6:**
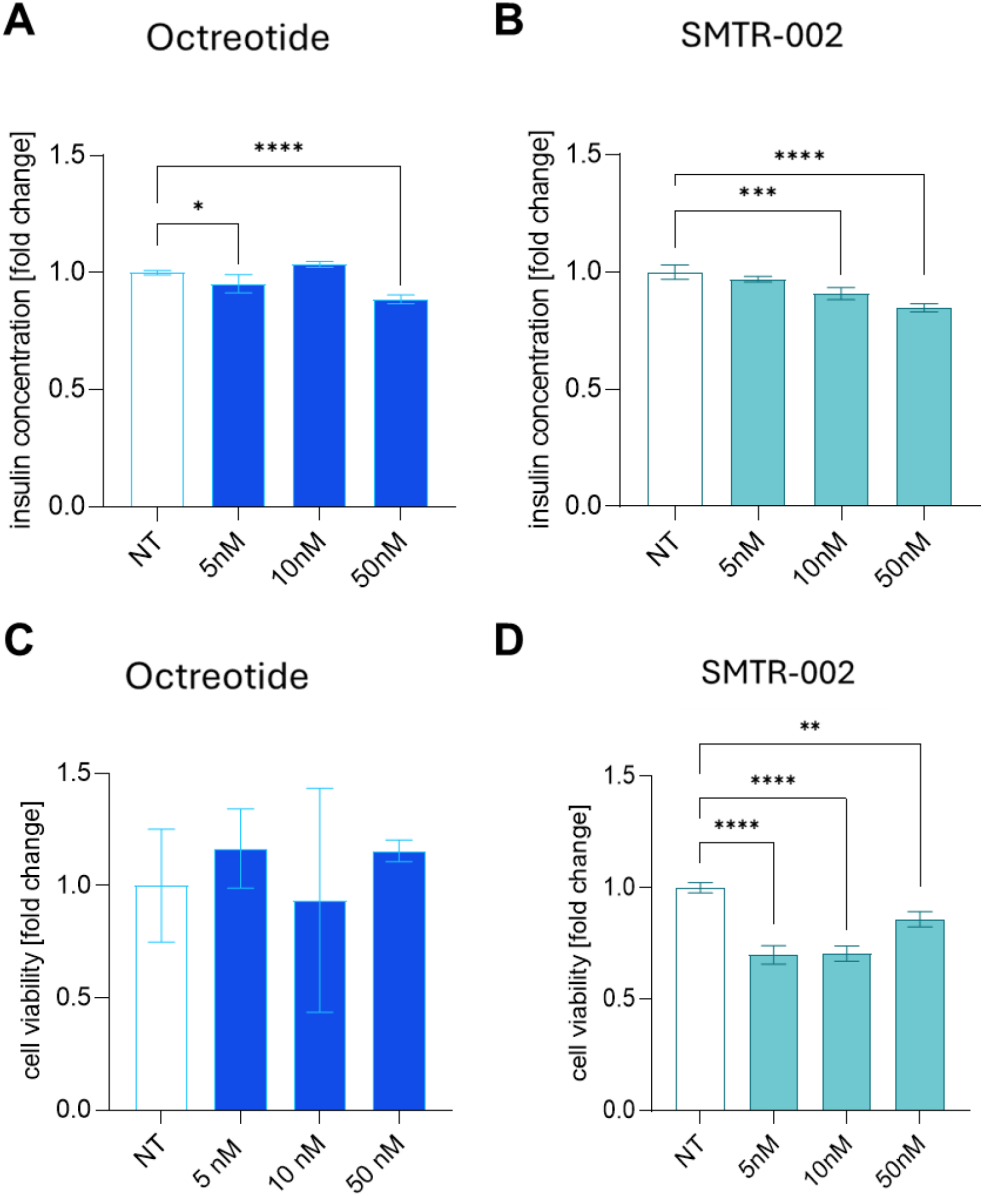
Effect of octreotide and SMTR-002 on insulin secretion and viability of NT-3 cells in the 3D culture model. **(A, B)** NT-3 cells were seeded at a density of 20, 000 cell/well in ULA plates and allowed to form spheres (5 days). Then they were treated with **(A)** octreotide or **(B)** the lead peptide (=SMTR-002) at the indicated concentrations (5nM, 10nM, 50nM). After 5 days, supernatants were collected, and insulin concentration quantified using the Human Insulin (INS) ELISA Kit (Elabscience). Data shown are the average of 3 technical replicates ± standard deviation (SD). NT, untreated. **(C, D)** In cultures parallel to panels A and B, Cell viability was carried out with CellTiter-Glo 3D assay. The experiment was repeated 3 times with similar results. Data shown are the average of 3 technical replicates ± SD. NT, untreated. Statistical analyses were carried out in GraphPad Prism using one-way ANOVA: *p ≤ 0.05; **p ≤ 0.01; ***p ≤ 0.001; ****p ≤ 0.0001.

The levels of insulin in the supernatant were measured after treatment with SMTR-002 or octreotide following previously optimized protocols (34). In these conditions, SMTR-002 elicited a dose-dependent decrease in insulin secretion (-3% to -15%), which was more potent than octreotide at the 10nM concentration (-9% for SMTR-002, p ≤ 0.001 *versus* untreated control; octreotide, n.s.) (**Figures 6A, B**). We then evaluated the effect of SMTR-002 on the proliferation of 3D organotypic cultures of NT-3 cells, and the results were compared with octreotide (**Figures 6C, D**). SMTR-002 significantly (p ≤ 0.01 to p ≤ 0.0001) decreased cell viability at all the doses tested when compared to untreated spheroids (from -14% to -30%) (**Figure 6D**). In contrast, octreotide did not significantly affect cell proliferation at the tested concentrations (**Figure 6C**).

## Discussion

In the current study, we present the first characterization of a new series of dual SSTR2/SSTR5 agonists in pituitary tumor and pancreatic NET, and we establish that these novel compounds are biologically active at low nanomolar concentrations and effectively inhibit both hormone production and tumor proliferation.

Studies of the AtT-20 mouse corticotroph cell line (an established model of ACTH-secreting pituitary tumors) revealed that four of the five novel dual SRLs (SMTR-001 to SMTR-004) markedly inhibited ACTH secretion and cell proliferation at low nanomolar concentrations. SMTR-002 and SMTR-003 achieved antiproliferative effects comparable or superior to those of pasireotide, while SMTR-001 and SMTR-004 exerted potent antisecretory effects similar to pasireotide’s profile. In parallel studies on the human insulinoma cell line NT-3, we demonstrated that the dual SRLs significantly reduce insulin secretion across a range of doses, with SMTR-002 and SMTR-003 exhibiting anti-secretory potency. At 10 nM, SMTR-002 and SMTR-004 inhibited insulin secretion to a significantly greater extent than octreotide. Importantly, the dual SRLs displayed significant antiproliferative effects, with SMTR-002 showing the strongest efficacy in reducing NT-3 cell proliferation. Collectively, these findings demonstrate that dual SSTR2/SSTR5 agonism can effectively suppress hormone secretion and tumor growth in both pituitary and pancreatic NET models.

After that initial testing of the five dual SRLs, SMTR-002 was selected as our lead peptide due to its equal and subnanomolar affinity for both SSTR2 and SSTR5, and for its good efficacy in both the AtT-20 corticotroph and NT-3 insulinoma cell models. This agent demonstrated potent inhibition of hormone secretion and cell proliferation at low doses (5 nM and 10 nM), outperforming other test compounds and even reference drugs like octreotide and pasireotide. Its consistent potency and favorable pharmacological profile make SMTR-002 a promising candidate for further preclinical development. With SMTR-002, we extended our analysis to 3D spheroid models of pancreatic NETs, which offer a more physiologically relevant system by mimicking the cell-cell interactions and architectural features of native tissues. SMTR-002 was highly effective in 3D NT-3 panNET spheroids, significantly reducing both insulin secretion and cell viability at all tested concentrations. These results support the use of 3D models in drug development, as they more accurately reflect the *in vivo* behavior of tumor cells and may provide better predictions of clinical outcomes. When comparing the ability of octreotide and SMTR-002 to suppress insulin secretion in 2D *versus* 3D culture systems, the suppression is greater (= lower hormone levels) in the former. This difference may partly reflect variations in treatment conditions as well as intrinsic differences between the two culture models. In 3D cultures, the complex spheroid architecture generates gradients of nutrients and oxygen, limiting drug penetration and reducing drug actions, particularly in the inner layers (38–40). Moreover, 3D models more closely reproduce cell-cell interactions and extracellular matrix components of the *in vivo* tumor microenvironment, which may modulate drug responsiveness and reduce cellular sensitivity to these compounds.

Finally, our study investigated the effect of SMTR-002 on intracellular signaling pathways, specifically cAMP modulation, in pituitary tumor cells. We found that SMTR-002 was highly effective at reducing forskolin-induced cAMP accumulation, with efficacy comparable to that of pasireotide and superior to that of octreotide. This suggests that the dual SSTR2/SSTR5 agonists exert their anti-secretory and anti-proliferative effects in pituitary tumor cells, at least in part, through cAMP pathway modulation, a well-established mechanism for SSTR activation in these cells (37).

The potential advantages of dual SSTR2/5 agonists are supported by a sizeable body of historical evidence. Initial studies in the field demonstrated the independent roles of SSTR2 and SSTR5 in regulating growth hormone (GH) and thyroid-stimulating hormone (TSH) secretion from the anterior pituitary (41) with subsequent work showing that combining specific compounds targeting SSTR2 and SSTR5 produced an additive inhibitory effect on GH secretion (42). This suggested that targeting both receptors could be beneficial in overcoming resistance to SSTR2-specific agents, such as in somatotropinomas (42). Later studies further reinforced this notion, showing that dual-affinity SRLs, such as the dual SSTR2/5 agonist BIM-23244, had greater GH inhibitory effects (43, 44). Additionally, it has been demonstrated that SSTR2 and SSTR5 can form heterodimers at the cell membrane, influencing receptor internalization and signaling in pituitary and other tumor models (45–49). Taken together, these findings indicate that SSTR2 and SSTR5 could have a synergistic role in regulating hormonal and proliferative effects, with dual receptor agonism providing an additive effect over single receptor targeting. The current study provides evidence for additional effects on proliferation in NET being possible using compounds that simultaneously target SSTR2 and SSTR5. Whether these effects in NT-3 cells are mediated through the same or different signaling pathways downstream of SSTR2 and SSTR5 remains to be established.

The clinical pharmacology of SRLs is a mature field that dates back to the early 1980’s. Over time, three compounds octreotide, lanreotide and pasireotide have become mainstream clinical therapeutics for pituitary adenomas and NETs. These compounds act as a yardstick for assessing the viability of new SRLs across the development process, beginning with receptor affinity profiles. Octreotide and lanreotide are closely related molecules and demonstrate high affinity only for SSTR2 (IC50 0.38-0.54 nM), while their affinities for SSTR5 are moderate (IC_50_: 6.3–17 nM) (50). In contrast, pasireotide has 2.5 times lower affinity for SSTR2 than octreotide and 40 times higher affinity at SSTR5; pasireotide also has low nanomolar affinity for SSTR1 (IC_50_: 9.3nM), and for SSTR3 (IC_50_: 1.5 nM), about 4.7-9.3 times that of octreotide. Among approved SRLs, therefore, the combination of octreotide-like activity at SSTR2 and pasireotide-like activity at SSTR5 is not present in any single compound. The current *in vitro* data appear consistent with the binding data of the novel compounds as potentially mediating potent antihormonal and antiproliferative effects through both SSTR2 and SSTR5. Whether these *in vitro* characteristics are therapeutically relevant will first require validation in preclinical *in vivo* studies, such as xenograft models bearing SSTR2- and SSTR5-positive tumors.

Other established pharmacological characteristics of SRLs are relevant for the design and interpretation of preclinical profiles of novel compounds. Given the pharmacological properties of SRLs, the lack of a classical dose-response curve observed in our experiments may be attributed to factors such as receptor saturation, internalization, and cellular tolerance. SSTR2 and SSTR5 receptors are high-affinity receptors that achieve near-maximal occupancy at low nanomolar concentrations and further increases in SRL concentration are unlikely to yield additional effects due to receptor desensitization and internalization (51, 52). This phenomenon is common with G-protein-coupled receptor (GPCR) agonists, where prolonged exposure can lead to a reduction in receptor availability and diminished signaling (53). In clinical practice, these challenges can be mitigated by optimizing dosing schedules, to minimize desensitization (54, 55). Additionally, combining SRLs with therapies targeting complementary pathways may help to enhance treatment efficacy (54, 56).

In clinical practice, long-acting depot formulations of octreotide and lanreotide are standard of care treatments for symptom control in functional NETs and have been shown to slow tumor progression in GEP-NETs (10). These SRLs are often initiated early in treatment and maintained even in the event of disease progression, with dose escalation and the addition of second-line therapies as needed. However, in the case of non-responders, no second-line SSA therapies are available for NET patients who fail to respond to octreotide or lanreotide. Unlike in acromegaly, where pasireotide offers an effective alternative, NET patients with progressive disease have no somatostatin-based medical therapeutic options. In a phase III study, pasireotide was not superior to octreotide in symptom control but showed better tumor control at six months in an interim analysis (57). However, when combined with everolimus, pasireotide did not improve progression-free survival in patients with advanced pancreatic NETs (58). A study program to assess the effect of continuing versus stopping octreotide/lanreotide therapy during second-line treatment of patients with progressive metastatic non-functional GEP NETs was recently announced (59). Our findings suggest that dual SRLs may offer a valuable alternative, potentially in the context of progressive or resistant NETs. In the direct comparison of SMTR-002 and octreotide in NT-3 cells, SMTR-002 exhibited more potent inhibition of insulin secretion and cell proliferation, especially at the 10 nM concentration, supporting the concept that superior efficacy over established treatment is possible. These results are promising for the clinical application of dual SSTR2/SSTR5 agonists in patients who have either failed current SSA treatments or have tumors that are resistant to SSTR2-targeted therapies.

The clinical potential of SRLs has been further reinforced by the development of radioligand therapies (RLTs), which target SSTRs for both diagnostic and therapeutic purposes. For example, PET/CT using ^68^Ga-DOTATATE, which specifically targets SSTR2, is widely used for diagnostic and staging purposes in NETs, while ^177^Lu-octreotate (Lutathera^®^), a SSTR2-specific radioligand, has proven to be effective in treating patients with SSTR2-positive tumors that are progressing despite treatment with octreotide. However, SSTR expression in NETs and other tumors is heterogeneous, with frequent expression of SSTR5 in addition to SSTR2 (60, 61). The incorporation of dual SSTR2/SSTR5 targeting agents, such as those we have evaluated in this study, could theoretically help enhance the delivery of radioligands to a broader range of tumor targets.

In conclusion, our study provides new preclinical evidence for the potential of dual SSTR2/SSTR5 agonists as a novel therapeutic approach for treating functional panNETs. These agents were able to reduce hormone secretion and tumor cell proliferation at least as effectively, and sometimes even more potently, than current SSA therapies. To advance these compounds toward clinical use, standard preclinical toxicology studies and *in vivo* efficacy testing in xenograft models of SSTR2- and SSTR5-expressing cells would be required, both as cold compounds and as potential components of novel RLT. Subsequent clinical trials would then be needed to evaluate their therapeutic potential in patients with metastatic NETs and other SSTR-positive tumors.

## Data Availability

We generated no datasets only experimental results. The raw data of our assays can be shared upon request. Requests should be directed to nataliasimona.pellegata@unipv.it.

## Supplementary material

Supplementary Figure 1 Validation of AtT-20 and NT-3 cells as experimental model. (A) RealTime RT-PCR for *SSTR2*, *SSTR3* and *SSTR5.* (B) Western blotting using anti-SSTR2 and anti-SSTR5 antibodies. GAPDH was used as reference control.

Supplementary Figure 2 Validation of ACTH secretion of AtT-20 cells in 2D. 3500 cells were plated in the presence or absence of FBS and ACTH secretion was assessed 72h later using a specific ELISA kit.

Supplementary Figure 3 NT-3 cells grown as 3D organotypic cultures. **(A)** Representative pictures of NT-3 cells grown in ULA pates for 14 days. **(B)** Insulin levels in the supernatant of NT-3 cells grown in 2D or in 3D. NT-3 cells were plated in regular plates (2D culture) or in ULA plates (3D spheroids). The supernatant was collected 5 days after plating for 2D cultures, or 5 days after 3D spheroid formation. Insulin levels were quantified in duplicate using an ELISA kit (Elabscience), following the manufacturer’s protocol.
