# Supplementary material for: Anti-secretory and anti-proliferative actions of next-generation dual subtype 2 and 5 somatostatin receptor ligands in neuroendocrine tumor models
Source: Front Oncol. 2026 Feb 27;16:1766563. doi: 10.3389/fonc.2026.1766563 (PMC12982089; doi:10.3389/fonc.2026.1766563)
Supplement: Supplementary Figure 1 — Validation of AtT-20 and NT-3 cells as experimental model. (A) RealTime RT-PCR for SSTR2, SSTR3 and SSTR5. (B) Western blotting using anti-SSTR2 and anti-SSTR5 antibodies. GAPDH was used as reference control. [file Image1.pdf]

SUPPLEMENTARY FIGURES

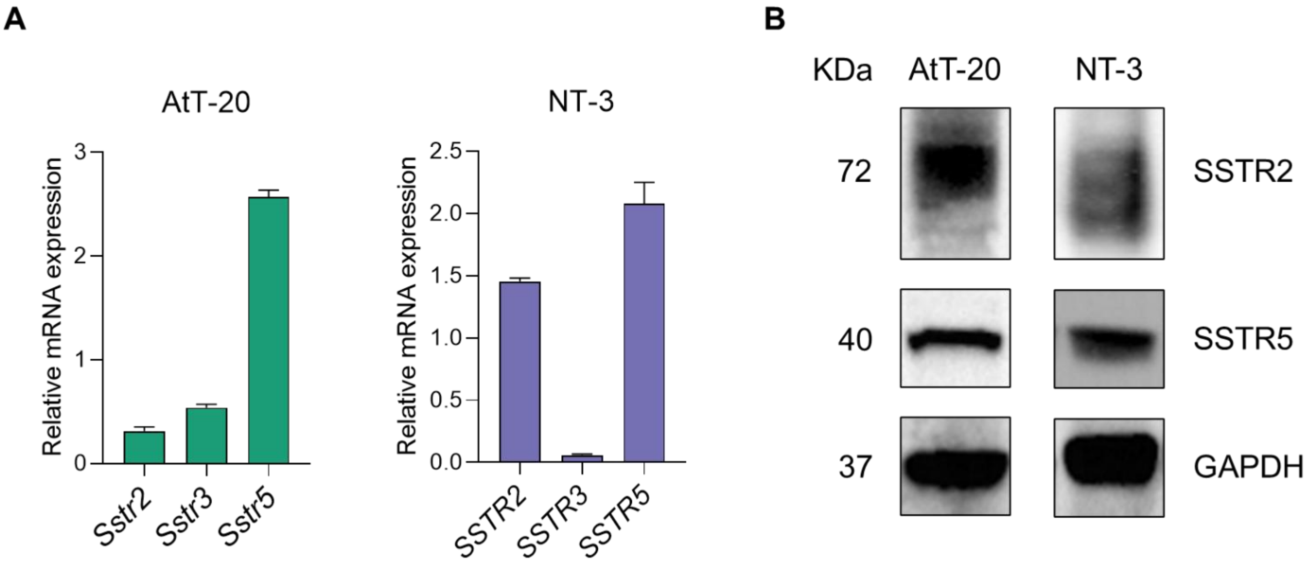

Supplementary Figure 1. Validation of AtT-20 and NT-3 cells as experimental model. (A) RealTime RT-PCR for *SSTR2*, *SSTR3* and *SSTR5.* (B) Western blotting using anti-SSTR2 and anti-SSTR5 antibodies. GAPDH was used as reference control.

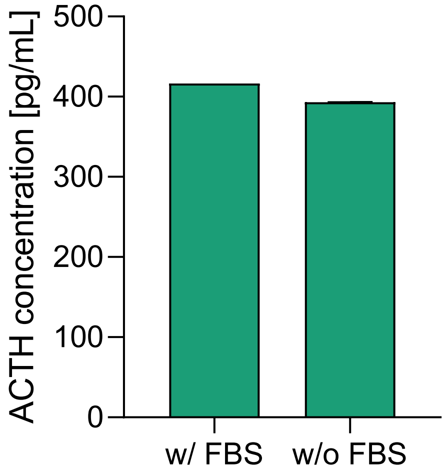

Supplementary Figure 2. Validation of ACTH secretion of AtT-20 cells in 2D. 3500 cells were plated in the presence or absence of FBS and ACTH secretion was assessed 72h later using a specific ELISA kit.

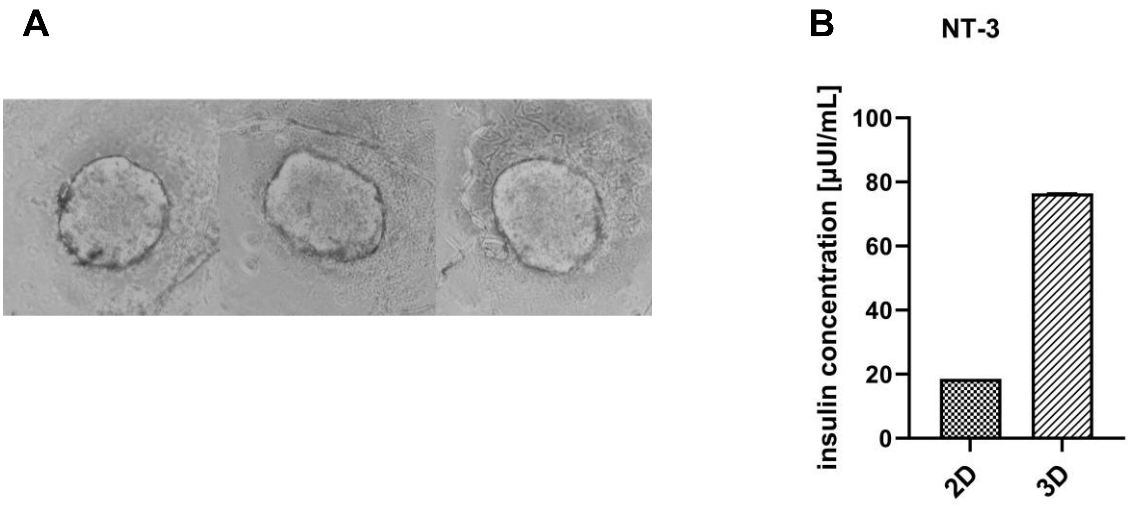

Supplementary Figure 3. NT-3 cells grown as 3D organotypic cultures. (A) Representative pictures of NT-3 cells grown in ULA pates for 14 days. (B) Insulin levels in the supernatant of NT-3 cells grown in 2D or in 3D. NT-3 cells were plated in regular plates (2D culture) or in ULA plates (3D spheroids). The supernatant was collected 5 days after plating for 2D cultures, or 5 days after 3D spheroid formation. Insulin levels were quantified in duplicate using an ELISA kit (Elabscience), following the manufacturer's protocol
